# Transfusion

**Published:** 1889-10-19

**Authors:** 


### THE PRACTITIONER'S OUTLOOK AND RETROSPECT.

### MEDICINE.

### TRANSFUSION.

Mr. Mayo Robson, honorary surgeon to the Leeds General Infirmary, records "Three cases of tranfusion."* The first case was hæmorrhage after delivery, the husband giving the blood. Mr. Mayo Robson thus describes the operation: "While I was exposing the vein of the patient, and introducing the pipette of an Aveling's transfuser into the median basilic vein, Mr. Jessop bled the donor to eight ounces, the blood being received into a clean warm basin, in which it was well whipped with a silver fork for a few minutes, and then filtered through fine muslin into a saline solution, made by mixing a small teaspoonful of salt with a tumbler full of warm water." This mixture, after being again passed through muslin, was injected gradually by means of the Aveling infuser, which had been previously filled with saline solution in order to avoid the possibility of air being forced into the circulation. On the third syringefull being injected, the patient, who had been unconscious even to not feeling the incision, opened her eyes, and in a dreamy manner said, "Oh! I begin to feel warm all over." About half a minute was allowed to elapse between each syringefull. After fourteen ounces had been injected the pipette was withdrawn, and a catgut ligature, which had been previously passed round the vein by means of an aneurism needle, was tightened and cut short. The patient was then perfectly conscious, had a fair colour in her lips, and expressed herself as feeling quite comfortable. Her recovery was uninterrupted.

The second transfusion was for hæmorrhage after miscarriage; a similar operation was performed, and recovery was uninterrupted, although slow.

The third instance was a female suffering from cancer of the uterus, which had been bleeding for some weeks. Colohysterectomy was performed and free bleeding occurred, which in her ex-sanguine condition rendered her pulseless. After the operation of transfusion the patient rallied for a time, but subsequently succumbed, life having apparently been prolonged for a few hours. Mr. Robson remarks that in the cases which have come under his notice where a saline infusion has been employed alone without admixture of blood, the relief has only been temporary. In the foregoing cases no special antiseptic measures were adopted. If in an emergency an Aveling's transfuser were not at hand, the author would extemporise a pipette from a fine glass tube, and attach to this a piece of rubber tubing, using as an injector an ordinary syringe, or even a small funnel.

* *Provincial Medical Journal*, August, 1889.

